# Enantioselectivity in the cytochrome P450-dependent conversion of tegafur to 5-fluorouracil in human liver microsomes

**DOI:** 10.1002/prp2.9

**Published:** 2013-10-23

**Authors:** Ikuo Yamamiya, Kunihiro Yoshisue, Yuji Ishii, Hideyuki Yamada, Ken-ichiro Yoshida

**Affiliations:** 1 Pharmacokinetics Research Laboratories, Taiho Pharmaceutical Co. Ltd.Tsukuba, Japan; 2 Graduate School of Pharmaceutical Sciences, Kyushu UniversityFukuoka, Japan

**Keywords:** 5-fluorouracil, cytochrome P450 2A6, enantioselectivity, microsomes, tegafur, thymidine phosphorylase

## Abstract

Tegafur (FT) is a prodrug of 5-fluorouracil (5-FU) used in cancer chemotherapy, and the bioactivation of FT to 5-FU is mainly catalyzed by cytochrome P450 (CYP) in hepatic microsomes. FT has a chiral center and is a racemate consisting of the enantiomers, *R*- and *S*-FT. In the present study, we clarified the enantioselectivity in the conversion of FT to 5-FU and identified human CYP isoforms involved in the metabolism of its enantiomers using human hepatic preparations and recombinant CYP isoforms. Although 5-FU was generated from both FT enantiomers, *R*-FT was a preferred substrate than *S*-FT, because of the considerably higher intrinsic clearance for 5-FU formation from *R*-FT in liver. Eadie–Hofstee plots in microsomes showed that the conversions of *R*- and *S*-FT to 5-FU followed biphasic and monophasic kinetics, respectively. Based on the evaluation using cDNA-expressed enzymes, CYP2A6 showed the highest activity for 5-FU formation from *R*-FT with the *K*_*m*_ value similar to that of the high-affinity component in microsomes. Also, CYP2A6 was the most effective catalyst for *S*-FT. Inhibition studies using CYP-selective inhibitors and anti-CYP antibodies demonstrated that CYP2A6 mainly contributed to the enantioselective metabolism of FT, and were almost in accordance with the relative percentage contribution of each CYP isoform to the metabolism of FT estimated using relative activity factor methods. These results suggest that the enantioselectivity in the bioactivation of FT to 5-FU in humans is mainly due to the large difference of the catalytic activity of CYP2A6 between *R*- and *S*-FT.

## Introduction

**Figure 1:**
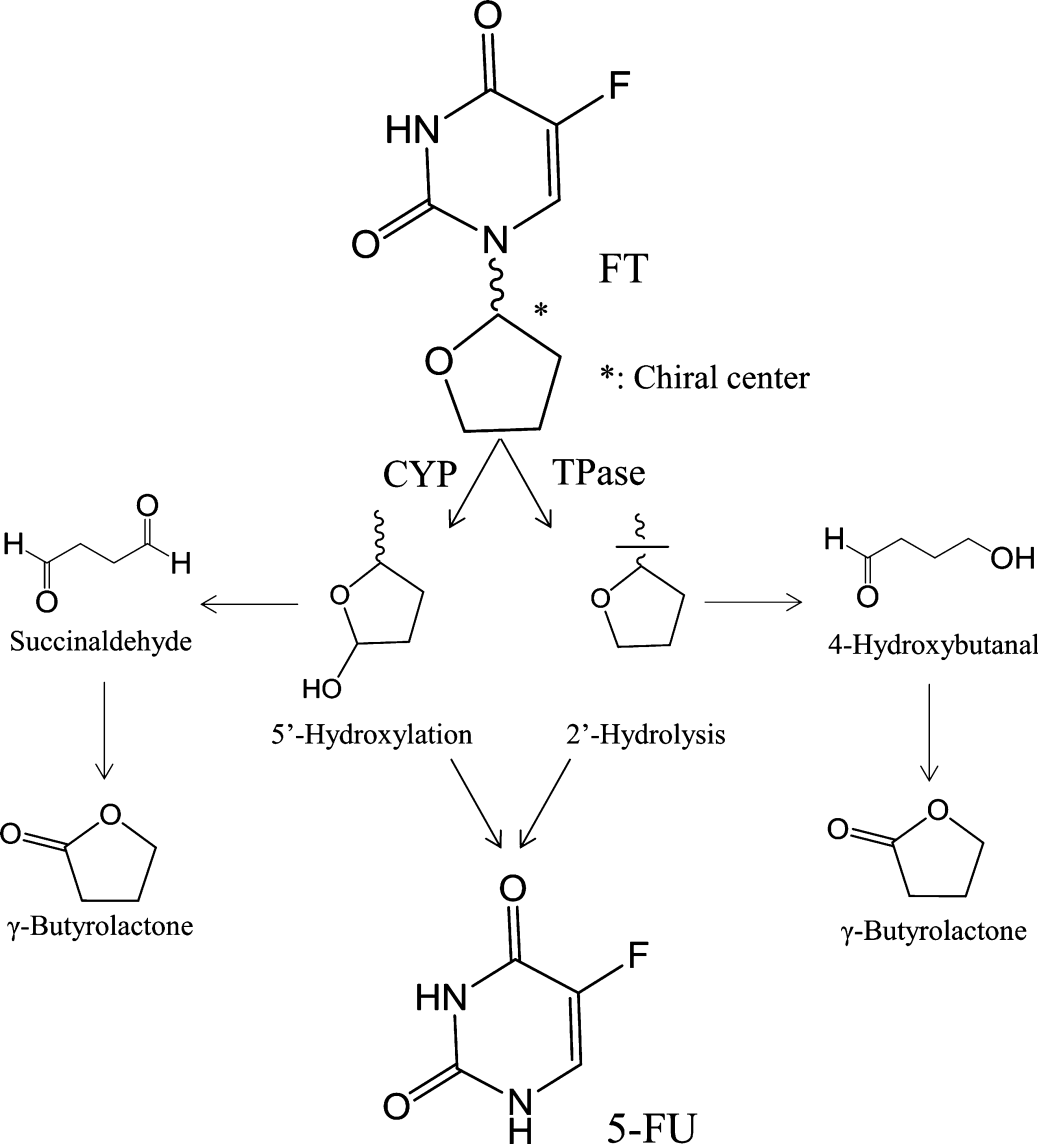
Pathway of the conversion of FT to 5-FU in liver.

Tegafur [5-fluoro-1-(2-tetrahydrofuryl)-2, 4 (1*H*, 3*H*)-pyrimidinedione; FT] is a prodrug of 5-fluorouracil (5-FU) and has been clinically used for cancer chemotherapy. Also, 5-FU has been used as an antitumor agent during the last five decades in the treatment of solid tumors. Previous reports have revealed that cytochrome P450 (CYP) and thymidine phosphorylase (TPase) in liver mainly catalyze the conversion of FT to its active form, 5-FU, in humans and animals (El Sayed and Sadée 1982, 1983; Ikeda et al. 2000; Komatsu et al. 2000, 2001; Kajita et al. 2003). Thus, these metabolic pathways are the main elimination routes of FT from the body. As shown in Figure 1, the tetrahydrofuran ring of FT is thought to undergo hydroxylation at its 5′-position catalyzed by CYP. This initial metabolism renders FT susceptible to the conversion to 5-FU, because the hydroxy metabolite of FT is chemically unstable (Lin et al. 1979). On the other hand, TPase catalyzes the hydrolytic cleavage at *N*1-*C*2′ bond of FT, which results in 5-FU formation (El Sayed and Sadée 1983). Our previous report has shown that CYP and TPase metabolize the tetrahydrofuran ring of FT to succinaldehyde and 4-hydroxybutanal, respectively, through the formation of the unstable intermediates during the metabolism of FT to 5-FU, followed by their sequential conversion to γ-butyrolactone, which possesses an antiangiogenesis effect (Fig. 1) (Yamamiya et al. 2010). FT is a chiral molecule and contains an asymmetric carbon at 2′-position of its tetrahydrofuran moiety as shown in Figure 1. In clinical practice, FT is administered as a racemic mixture of the enantiomers, *R*-FT and *S*-FT. In general, enantiomers show different biological properties, for example, pharmacodynamic effects or pharmacokinetic profiles because of their distinct affinity with target proteins. Regarding the in vitro enantioselective metabolism of FT, it has been reported that mouse and rabbit hepatic preparations are capable of preferentially catalyzing the cleavage of 2′-position of the tetrahydrofuran ring of *R*-FT, but not the hydroxylation of 5′-position followed by the decomposition to 5-FU, and both enantiomers are converted to 5-FU to a similar extent (Au and Sadée 1981). A previous study showed that the plasma concentration of *R*-FT was lower than *S*-FT in cancer patients after oral administration of tegafur-uracil (UFT), a combination drug consisting of uracil and racemic FT at a molar ratio of 4:1 (Damle et al. 2001). It is conceivable that the enantioselectivity observed in the in vivo pharmacokinetic profiles of FT in humans is due to the enantioselective conversion of FT to 5-FU in the liver because this metabolic process is an important factor determining body clearance. However, the enantioselective conversion of FT to 5-FU in the human liver remains poorly characterized, and thereby the relative contribution of each isomer to the therapeutic efficacy is unclear. In this study, therefore, we clarified the enantioselectivity in the conversion of FT to 5-FU in humans. We investigated the potential difference of kinetic profiles for 5-FU formation from the FT enantiomers and the involvement of CYP isoforms using human hepatic preparations and cDNA-expressed CYP isoforms.

## Material and Methods

### Chemicals

Enantiomers of FT, 5-chloro-2, 4-dihydroxypyridine (CDHP), 5-chloro-6-(2-iminopyrrolidin-1-yl) methyl-2, 4 (1*H*,3*H*)-pyrimidinedione hydrochloride (TPI), and ^*15*^*N*_*2*_-5-FU were synthesized at Taiho Pharmaceutical Co., Ltd. (Saitama, Japan). 1-Aminobenzotriazole was purchased from Tokyo Chemical Industry Co. (Tokyo, Japan). 5-FU, glucose-6-phosphate, furafylline, tranylcypromine hydrochloride, ticlopidine hydrochloride, phenacetin, acetaminophen, *S*-mephenytoin, and chlorzoxazone were purchased from Sigma-Aldrich Chemical Co. (St. Louis, MO). Magnesium chloride hexahydrate, sodium diethyldithiocarbamate, coumarin, and 7-hydroxycoumarin were purchased from Wako Pure Chemical Industries (Osaka, Japan). β-NADP^+^ and glucose- 6-phosphate dehydrogenase were purchased from Oriental Yeast Co. (Tokyo, Japan). 4′-Hydroxymephenytoin and 6-hydroxychlorzoxazone were purchased from BD Gentest (San Jose, CA). Polyclonal antibodies against human CYP1A2, 2A6, 2C19, and 2E1 were purchased from Nosan Corporation (Kanagawa, Japan). Other chemicals used were of the highest grade available commercially.

### Human hepatic preparations and cDNA-expressed CYP isoforms

Human hepatic 9000*g* supernatant (S9) and cytosolic fraction were purchased from XenoTech, LLC. (pooled preparations from 20 donors; Kansas City, KS). Human hepatic microsomes from five individuals (HG32, HH94, HH81, HH74, and HH31), a pooled preparation from 150 donors, and microsomes prepared from insect cell lines expressing CYP1A2, 2A6, 2B6, 2C8, 2C9, 2C19, 2D6, 2E1, and 3A4 were purchased from BD Gentest. Insect microsomes expressing only NADPH-P450 oxidoreductase and cytochrome b5 were also used as control. These enzymes were stored at −80°C until use.

### Assay of 5-FU formation from FT

5-FU formed from FT was subjected to extensive metabolism by dihydropyrimidine dehydrogenase (DPD) contaminating human hepatic preparations. Therefore, a potent DPD inhibitor, CDHP, was always added to avoid the unexpected loss of 5-FU (Ikeda et al. 2000; Yamamiya et al. 2010). Incubation mixture for hepatic microsomal metabolism contained FT, microsomes (1 mg protein/mL), 0.1 mmol/L CDHP, and an NADPH-generating system consisting of 1.3 mmol/L β-NADP^+^, 3.3 mmol/L glucose-6-phosphate, 3.3 mmol/L magnesium chloride, and 0.4 units glucose-6-phosphate dehydrogenase in 100 mmol/L Tris (pH 7.4). Metabolic reaction by S9 and cytosol (each 2 mg protein/mL) was carried out in 100 mmol/L phosphate (pH 7.4) because TPase requires phosphate ions for catalyzing the reaction. In some cases, insect microsomes expressing recombinant CYP isoforms (20 pmol/mL) were added to the incubation mixtures in either 50, 100 mmol/L phosphate (pH 7.4) or 100 mmol/L Tris (pH 7.4), depending on supplier's recommendation. Microsomal protein concentrations of all cDNA-expressed CYPs were adjusted to 0.5 mg protein/mL by adding control microsomes expressing NADPH-P450 oxidoreductase and cytochrome b5. The reaction for the assay of FT metabolism was initiated by adding the substrate, following preincubation for 5 min at 37°C. After incubation at 37°C, the reaction was stopped by adding three volumes of ice-cold acetonitrile. The incubation times of microsomes, cytosol, and S9 were 15, 15, and 30 min, respectively. After centrifugation, the supernatant was collected and stored at −80°C until the determination of 5-FU. Because a small portion of FT is nonenzymatically converted to 5-FU, the content of 5-FU spontaneously formed was subtracted from total yield obtained after incubation to correct the activity. The spontaneous degradation of FT to 5-FU was evaluated using enzymes inactivated by heating them at 100°C for 5 min.

### Inhibition study

The effects of inhibitors of CYP isoforms and TPase, and anti-CYP antibodies on 5-FU formation from FT enantiomers catalyzed by human hepatic preparations were evaluated. In inhibition assays, FT enantiomers were used at the concentrations of 30 μmol/L. 1-Aminobenzotriazole (1 mmol/L) and TPI (10 μmol/L) were used as nonselective inhibitors of CYP isoforms and TPase, respectively. To evaluate the contributions of CYP isoforms to the enantioselective metabolism of FT, furafylline (25 μmol/L), tranylcypromine (5 μmol/L), ticlopidine (20 μmol/L), quinidine (1 μmol/L), diethyldithiocarbamate (100 μmol/L), and ketoconazole (1 μmol/L) were used as CYP-selective inhibitors for CYP1A2, CYP2A6, CYP2C19, CYP2D6, CYP2E1, and CYP3A, respectively. Inhibitors were dissolved in methanol and diluted with 100 mmol/L phosphate (pH 7.4) or 100 mmol/L Tris (pH 7.4) so that the final concentration of solvent in the incubation mixture was 0.5%. Methanol was also added to the control at the same concentration as the conditions with inhibitors. Inhibitory effects of anti-CYP antibodies were examined by preincubating microsomes with the antibodies for 10 min on ice. Each polyclonal anti-CYP antibody was used at a concentration ranging 10–40 μL/mg microsomal protein according to the procedure recommended by the supplier. The reaction was performed similarly as described above, except for evaluating the inhibitory effect of furafylline. Because furafylline is a potent mechanism-based inhibitor for CYP1A, the enantioselective metabolism of FT was initiated, following the preincubation of microsomes with furafylline in the presence of an NADPH-generating system for 20 min at 37°C.

### Quantification of 5-FU

Concentration of 5-FU was determined using a LC/MS/MS system. The analytical system consisted of a HP1100 liquid chromatograph (Agilent Technologies, CA) coupled with an API4000 triple-quadrupole mass spectrometer (Applied Biosystems, CA) equipped with Turbo V source and ESI interface. Sample separation was performed using an Unison UK-Amino column (2.0 mm i.d. × 100 mm, 3 μm; Imtakt, Kyoto, Japan) at a flow rate of 0.2 mL/min at 40°C. The mobile phase consisted of 10 mmol/L ammonium acetate/acetonitrile 10:90 (v/v). The MS/MS analysis was performed in negative ionization mode under multiple reaction monitoring (MRM) mode, using mass transitions, m/z 128.6/41.8 for 5-FU and m/z 130.6/42.8 for ^*15*^*N*_*2*_-5-FU, an internal standard (IS). To 100 μL of the supernatant obtained after metabolic incubation, 50 μL of 75% acetonitrile containing 500 ng/mL IS was added, and then 5 μL of the mixture was injected into the LC/MS/MS system. For quantification of 5-FU, the calibration curves were linear over the range 2–1000 ng/mL.

### Estimation of contributions of CYP isoforms to the enantioselective metabolism of FT

To calculate relative activity factor (RAF) that estimates the contribution of the CYP isoform to a particular metabolic reaction, metabolite formation rates for CYP1A2-catalyzed phenacetin *O*-dethylation, CYP2A6-catalyzed coumarin 7-hydroxylation, CYP2C19-catalyzed *S*-mephenytoin 4′-hydroxylation, and CYP2E1-catalyzed chlorzoxazone 6-hydroxylation were determined with human hepatic microsomes and cDNA-expressed CYP isoforms. Stock solutions of phenacetin, coumarin, *S*-mephenytoin, and chlorzoxazone were prepared in acetonitrile and added to the incubation mixtures at much lower concentrations than their apparent *K*_*m*_ values reported (<10-fold). The reaction mixture contained a CYP-selective substrate, an NADPH-generating system, and human hepatic microsomes (0.2 mg protein/mL) or cDNA-expressed CYP (20 pmol/mL) in adequate buffer for the CYP isoform. The reaction was initiated by adding an NADPH-generating system, followed by incubation for 5 to 25 min at 37°C. After stopping the reaction by adding three volumes of ice-cold acetonitrile containing IS, the incubation mixture was centrifuged and the supernatant was evaporated under a gentle nitrogen stream. The resultant residue was dissolved in mobile phase and subjected to measurement of the metabolites. The RAF was calculated based on the metabolic activity obtained using hepatic microsomes and recombinant CYP isoforms as the enzyme sources (see the later section for the details). The yields of CYP isoform-specific metabolites were determined using the same instrument system as described before. The analytes were separated using an Inertsil ODS-3 column (2.0 mm i.d. × 150 mm, 3 μm; GL Sciences Inc., Tokyo, Japan). The column was eluted by a gradient consisting of 0.1% formic acid/water (mobile phase A) and acetonitrile (mobile phase B) at a flow rate of 0.2 mL/min at 40°C: 5–95% B in 5 min; 95–5% B in 0.1 min; 5% B hold for 4 min. The analytes were detected by either positive or negative ion spray in MRM mode: positive: acetaminophen, 7-hydroxycoumarin, and 4′-hydroxymephenytoin; negative: 6-hydroxychlorzoxazone. The precursor/product ions mass transitions were monitored for determination of the metabolites and ISs: m/z 152.0/110.0 for acetaminophen; m/z 163.0/107.0 for 7-hydroxycoumarin; m/z 235.0/150.0 for 4′-hydroxymephenytoin; m/z 184.0/120.0 for 6-hydroxychlorzoxazone; m/z 260.0/116.0 for propranolol (IS for the positive ion mode); m/z 240.0/196.0 for mefenamic acid (IS for the negative ion mode). The linearity of the calibration curves for all analytes were confirmed between 2 and 1000 ng/mL.

### Kinetic analysis

Kinetic studies were performed using microsomes from five human livers (HG32, HH94, HH81, HH74, and HH31), cDNA-expressed CYP isoforms, or pooled human hepatic cytosol. In determining kinetic parameters, FT concentration ranged from 0.03 to 10 mmol/L. All reactions were performed in a linear range of 5-FU formation with respect to protein or CYP concentrations and incubation time. Eadie–Hofstee plot analysis was used to characterize enzyme kinetics in which kinetic parameters were estimated by fitting the data to the following equations (1) or (2):

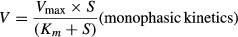
(1)

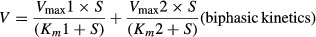
(2)where *V* is 5-FU formation rate, *S* is FT concentration in the incubation mixture, *K*_*m*_1 and *K*_*m*_2 are affinity constants for high- and low-affinity components, respectively, and *V*_max_1 and *V*_max_2 are maximum velocities for high- and low-affinity components, respectively. Kinetic parameters (apparent *K*_*m*_ and *V*_max_) were calculated using a computer program designed for nonlinear regression analysis (MULTI; Yamaoka et al. 1981).

### Calculation of RAF

Intrinsic clearance (CLint) was calculated as a ratio of V/S where V and S were the metabolite formation rate and the concentration of a probe substrate for CYP isoform, respectively, when the substrate concentration was well below the *K*_*m*_ value. The RAF for each CYP isoform was calculated according to equation (3) (Venkatakrishnan et al. 2000).

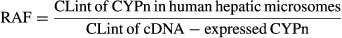
(3)n = 1A2, 2A6, 2C19, or 2E1.

The contribution of CYPn to the metabolism of *R*- and *S*-FT at their concentration of 30 μmol/L was subsequently calculated using equation (4).

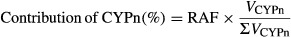
(4)*V*_CYPn_ = 5-FU formation rate in cDNA-expressed CYPn.

### Statistical analysis

Experiments were performed in triplicate and the data expressed as the mean ± standard deviation (SD), except for 5-FU formation rate catalyzed by individual human hepatic microsomes. The statistical significance was analyzed by the Student's *t*-test or Dunnet's test. Statistical Analysis System, version 6.12 (SAS Institute, Inc., Cary, NC) was used for all the statistical analyses.

## Results

### Enantioselective metabolism of FT in human hepatic S9

**Figure 2:**
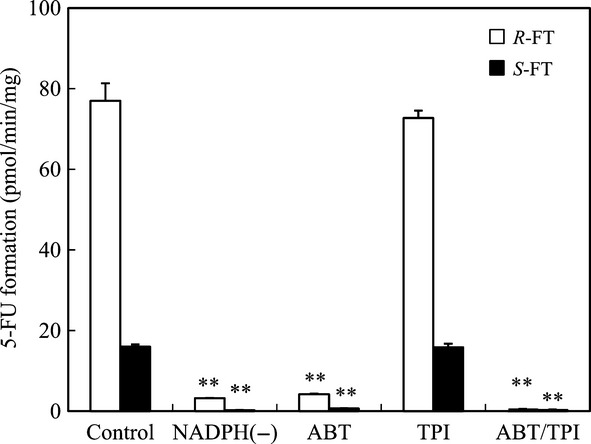
The enantioselective conversion of FT to 5-FU catalyzed by human hepatic S9. The enantiomers of FT (0.1 mmol/L) were incubated in human hepatic S9 (2 mg protein/mL) with an NADPH-generating system and 0.1 mmol/L CDHP at 37°C for 30 min in the absence or presence of each inhibitor. Inhibitors tested were 1-aminobenzotriazole (ABT, 1 mmol/L) and TPI (10 μmol/L). Opened and closed bars represent 5-FU formation rate from *R*- and *S*-FT, respectively. Data are presented as mean ± SD, *n* = 3. **Significantly different (*P* < 0.01) from the control.

Initially, we examined the enantioselective conversion of FT to 5-FU using human hepatic S9 with an NADPH-generating system to investigate the contributions of microsomal CYP and cytosolic TPase to this metabolic activation. We observed a linear and time-dependent increase in 5-FU formation from each FT enantiomer, and chiral inversion did not occur during the metabolic incubations (data not shown). The specific activity for 5-FU formation from FT clearly showed stereoselectivity, and human hepatic S9 favored *R*-FT than *S*-FT as the substrate (Fig. 2). Because the omission of an NADPH-generating system almost abolished 5-FU formation from both FT enantiomers, CYP was thought to play a major role in FT metabolism. Indeed, 1-aminobenzotriazole (1 mmol/L), a potent nonspecific CYP inhibitor (Emoto et al. 2003), markedly reduced the amount of 5-FU formed from both enantiomers to a similar level as the condition without NADPH, whereas TPI (10 μmol/L), a potent TPase inhibitor (Fukushima et al. 2000), had only a minor effect. A combination of both inhibitors resulted in complete inhibition against the enantioselective conversion of FT to 5-FU.

### Enzyme kinetic analysis for 5-FU formation from *R*- and *S*-FT in human hepatic preparations

**Figure 3:**
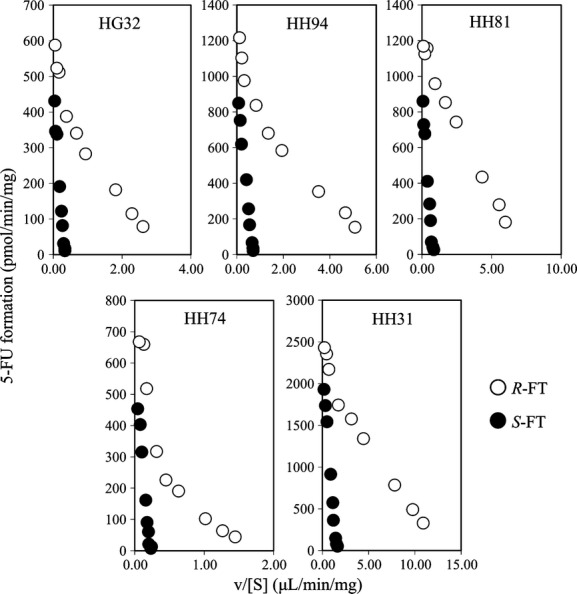
Eadie–Hofstee plots for the enantioselective conversion of FT to 5-FU catalyzed by microsomal preparations from five human livers. The enantiomers of FT (0.03–10 mmol/L) were incubated in human hepatic microsomes (1 mg protein/mL) with an NADPH-generating system and 0.1 mmol/L CDHP at 37°C for 15 min. Opened and closed circles represent 5-FU formation rate from *R*- and *S*-FT, respectively. Data points are the mean values (*n* = 2).

To characterize the conversion of *R*- and *S*-FT to 5-FU in more detail, we then determined the kinetic parameters in the reaction mediated by hepatic microsomes prepared from five donors. Eadie–Hofstee plots for 5-FU formation from *R*- and *S*-FT (0.03–10 mmol/L) are shown in Figure 3. The plots showed that 5-FU formation from *R*-FT in all preparations except HH81 took place in a biphasic manner, which suggests the involvement of multiple CYP isoforms. On the other hand, the metabolism of *S*-FT exhibited monophasic kinetics. The kinetic parameters for 5-FU formation from FT enantiomers are listed in Table 1. The high-affinity phase for *R*-FT metabolism exhibited the *K*_*m*_ value of less than 1/20 of that of low-affinity phase, whereas there was little difference in the *V*_max_ values between both phases. Thus, the high-affinity component showed the approximately 27-fold higher CLint (*V*_max_/*K*_*m*_) compared with the low-affinity one. The *K*_*m*_ value for *S*-FT was comparable to that in the low-affinity phase for *R*-FT, which suggests that *S*-FT only has a low-affinity clearance pathway. Regarding the enantioselective metabolism of FT in pooled human hepatic cytosol, 5-FU formation from *R*-FT, but not *S*-FT was observed. However, the CLint value of *R*-FT (0.029 μL min^−1^ mg^−1^; the apparent *K*_*m*_ and *V*_max_ values of 26 mmol/L and 761 pmol min^−1^ mg^−1^, respectively) was much lower compared with microsomes (figure and table not shown).

**Table 1:** The kinetic parameters for the enantioselective conversion of FT to 5-FU catalyzed by microsomal preparations from five human livers

Kinetic parameters	HG32	HH94	HH81	HH74	HH31	Mean ± SD
*R*-FT						
*V*_max_1 (pmol min^−1^ mg^−1^)	355	712	1162	146	1816	838 ± 669
*K*_*m*_1 (mmol/L)	0.11	0.12	0.16	0.093	0.14	0.12 ± 0.03
CLint1 (μL min^−1^ mg^−1^)	3.2	6.1	7.1	1.6	13	6.2 ± 4.4
*V*_max_2 (pmol min^−1^ mg^−1^)	302	583	–1	709	859	613 ± 236
*K*_*m*_2 (mmol/L)	3.0	2.3	–1	2.7	2.9	2.7 ± 0.3
CLint2 (μL min^−1^ mg^−1^)	0.10	0.26	–1	0.26	0.30	0.23 ± 0.09
CLint (μL min^−1^ mg^−1^)	3.3	6.4	7.1	1.8	13	6.4 ± 4.4
*S*-FT						
*V*_max_ (pmol min^−1^ mg^−1^)	468	936	915	583	2205	1021 ± 692
*K*_*m*_ (mmol/L)	1.4	1.3	1.1	2.6	1.4	1.6 ± 0.6
CLint (μL min^−1^ mg^−1^)	0.34	0.71	0.81	0.23	1.6	0.73 ± 0.53

The kinetic parameters were calculated from the fitted curves obtained by nonlinear regression analysis with MULTI. Mean (±SD) is the average of kinetic parameters obtained using five human preparations (HG32, HH94, HH81, HH74, and HH31).

1 HH74 exhibited a monophasic behavior for *R*-FT metabolism.

### Enantioselective conversion to 5-FU from FT by cDNA-expressed CYP isoforms

**Figure 4:**
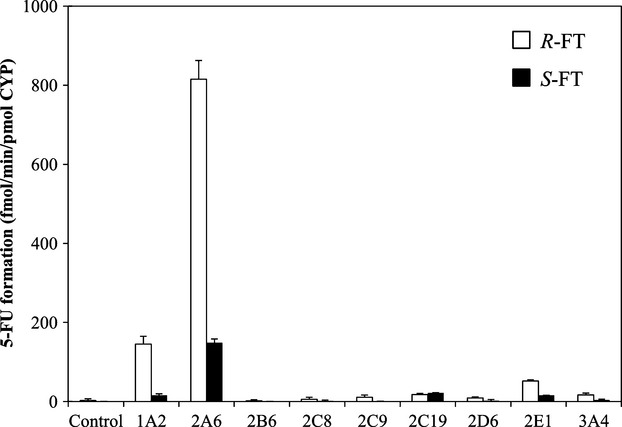
The enantioselective conversion of FT to 5-FU catalyzed by cDNA-expressed CYP isoforms. The enantiomers of FT (0.03 mmol/L) were incubated in recombinant human CYP1A2, 2B6, 2C8, 2C9, 2C19, 2D6, 2E1, and 3A4 (20 pmol CYP/mL) with an NADPH-generating system and 0.1 mmol/L CDHP at 37°C for 15 min. Opened and closed bars represent 5-FU formation rate from *R*- and *S*-FT, respectively. Data are presented as mean ± SD, *n* = 3.

To identify CYP isoforms preferentially catalyzing the metabolism of *R*- and *S*-FT to 5-FU, we evaluated the enzymatic activity using recombinant human CYP isoforms expressed in baculovirus–insect cells system coexpressing human NADPH-P450 oxidoreductase. As shown in Figure 4, CYP2A6 showed the highest activity in converting *R*-FT to 5-FU, followed by CYP1A2 and CYP2E1. Likewise, CYP2A6 showed preference for 5-FU formation from *S*-FT, and CYP1A2, 2C19, and 2E1 seemed to be involved in the metabolism to a lesser extent compared with CYP2A6. Other isoforms showed negligible activity for the enantioselective metabolism of FT. The kinetic parameters for 5-FU formation from *R*- and *S*-FT in recombinant CYP isoforms are summarized in Table 2. CYP2A6 exhibited the lowest *K*_*m*_, giving the highest CLint among four CYP isoforms toward both *R*- and *S*-FT. The rate of 5-FU formation catalyzed by CYP2C19 was increased linearly up to the concentration of 10 mmol/L *R*-FT; the apparent *K*_*m*_ value was calculated to be more than 10 mmol/L. Similar result was observed in the metabolism of *S*-FT catalyzed by CYP1A2.

**Table 2:** The kinetic parameters for the enantioselective conversion of FT to 5-FU catalyzed by cDNA-expressed CYP isoforms

Kinetic parameters	CYP1A2	CYP2A6	CYP2C19	CYP2E1
*R*-FT				
*V*_max_ (pmol min^−1^ pmol^−1^ CYP)	5.6 ± 0.3	4.5 ± 0.1	N.D.	8.4 ± 0.6
*K*_*m*_ (mmol/L)	1.1 ± 0.1	0.17 ± 0.01	>10	3.6 ± 0.3
CLint (nL min^−1^ pmol^−1^ CYP)	4.9	26	N.D.	2.3
*S*-FT				
*V*_max_ (pmol min^−1^ pmol^−1^ CYP)	N.D.	5.6 ± 0.3	5.5 ± 0.2	4.7 ± 0.2
*K*_*m*_ (mmol/L)	>10	1.1 ± 0.1	6.2 ± 0.3	3.7 ± 0.2
CLint (nL min^−1^ pmol^−1^ CYP)	N.D.	4.9	0.90	1.3

The kinetic parameters were calculated from the fitted curves obtained by nonlinear regression analysis with MULTI. N.D.: Not determined.

### Effects of CYP inhibitors and antibodies on the enantioselective conversion of FT to 5-FU

**Figure 5:**
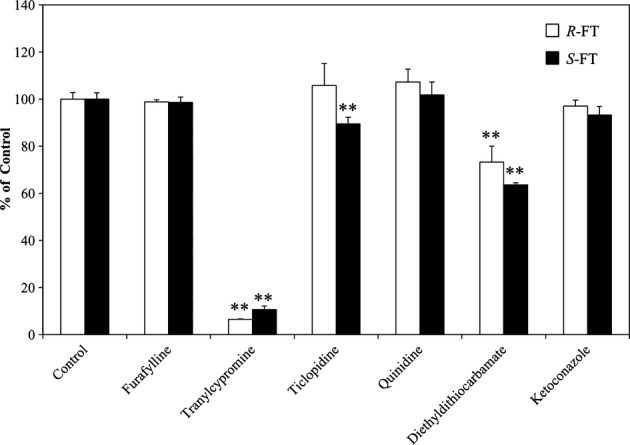
Inhibitory effects of CYP inhibitors on 5-FU formation from FT enantiomers catalyzed by human hepatic microsomes. The enantiomers of FT (0.03 mmol/L) were incubated in human hepatic microsomes (1 mg protein/mL) with an NADPH-generating system and 0.1 mmol/L CDHP at 37°C for 15 min in the absence or presence of each inhibitor. Inhibitors tested were furafylline (CYP1A2; 25 μmol/L), ticlopidine (CYP2C19; 20 μmol/L), tranylcypromine (CYP2A6; 5 μmol/L), quindine (CYP2D6; 1 μmol/L), diethyldithiocarbamate (CYP2E1; 100 μmol/L), and ketoconazole (CYP3A4; 1 μmol/L). For evaluating the effect of furafylline, the preincubation of microsomes with an inhibitor and an NADPH-generating system for 20 min was performed, followed by the enantioselective metabolic reaction. Opened and closed bars represent 5-FU formation rate from *R*- and *S*-FT, respectively. Data are presented as mean ± SD, *n* = 3. **Significantly different (*P* < 0.01) from the respective control (*R*- or *S*-FT) without inhibitor.

**Figure 6:**
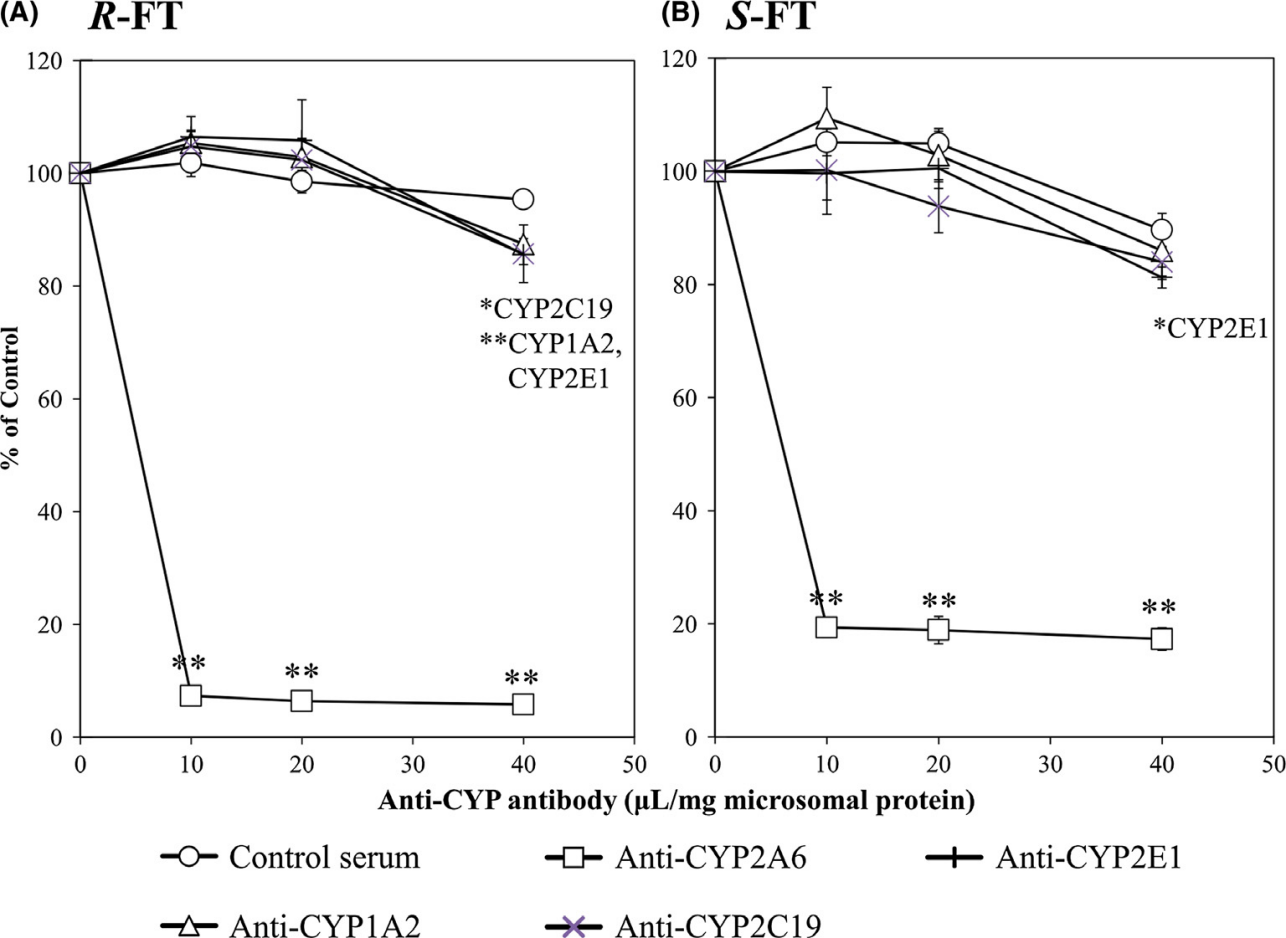
Inhibitory effects of anti-CYP antibodies on 5-FU formation from *R*- (A) and *S*-FT (B) catalyzed by human hepatic microsomes. The enantiomers of FT (0.03 mmol/L) were incubated in human hepatic microsomes (1 mg protein/mL) with an NADPH-generating system and 0.1 mmol/L CDHP at 37°C for 15 min in the presence of each anti-CYP antibody or preimmune serum. The concentration of preimmune serum, anti-CYP1A2, 2A6, 2C19, and 2E1 antibodies tested ranged 10–40 μL/mg microsomal protein. Data points are presented as mean ± SD, *n* = 3. **Significantly different (*P* < 0.01) from the control (addition of preimmune serum) at each end point.

To estimate the contributions of CYP isoforms to the enantioselective metabolism of FT, we next investigated the effects of CYP inhibitors and anti-CYP antibodies on the formation rate of 5-FU mediated by pooled human hepatic microsomes. Each inhibitor was used at a much higher concentration than its Ki value reported so far (Bourrié et al. 1996; Ko et al. 2000; Zhang et al. 2001; Baranová et al. 2005). Among six inhibitors tested, tranylcypromine (5 μmol/L), a potent CYP2A6 inhibitor, markedly inhibited 5-FU formation from both isomers by ∼90% (Fig. 5). The amounts of 5-FU formed from *R*- and *S*-FT were decreased to 73 and 64% of the control value, respectively, by diethyldithiocarbamate (100 μmol/L) used as a CYP2E1 inhibitor. Although ticlopidine (20 μmol/L), a CYP2C19 inhibitor, slightly reduced 5-FU formation from *S*-FT, furafylline (25 μmol/L), a CYP1A2 inhibitor, had no effect on the enantioselective metabolism of FT. Also, quinidine (a CYP2D6 inhibitor) and ketoconazole (a CYP3A4 inhibitor) had no effect. As can be seen in Figure 6, anti-CYP2A6 antibody caused more than 90 and 80% inhibitions against 5-FU formation from *R*- and *S*-FT, respectively, whereas anti-CYP1A2, 2C19, and 2E1 antibodies had little inhibitory effect on 5-FU formation from both enantiomers.

### Estimation of the contributions of CYP isoforms to the enantioselective conversion of FT to 5-FU using RAF approach

Table 3 summarizes the CLint values of probe reactions catalyzed by CYP1A2, 2A6, 2C19, and 2E1. Using these values, RAF for each CYP was calculated, and then applied for the estimation of the relative contribution (%) of a particular CYP to 5-FU production from FT (Table 3) (eqs. 3 and 4; see the Material and Methods). The contribution of CYP2A6 to 5-FU formation from both *R*- and *S*-FT was close to 90% (Table 3). In contrast, other CYP isoforms showed much lower contribution to the enantioselective metabolism of FT compared with CYP2A6.

**Table 3:** CLint, RAF for CYP isoforms, and contributions (%) to the enantioselective conversion of FT to 5-FU

CYP isoform (reported *K*_*m*_ value)	Metabolism of probe substrates			5-FU formation from FT			
	CLint (μL min^−1^ mg^−1^) hepatic microsomes	CLint (nL min^−1^ pmol^−1^ CYP) recombinant CYP isoforms	RAF	Velocity (pmol min^−1^ pmol^−1^ CYP)		CYP contribution (%)	
				*R*-FT	*S*-FT	*R*-FT	*S*-FT
CYP1A2 (30 μmol/L)^1^	151 ± 3	1.93 ± 0.08	78.2	145 ± 20	16.0 ± 3.4	7.5	4.4
CYP2A6 (4.4 μmol/L)^2^	860 ± 4	5.28 ± 0.11	163	815 ± 48	149 ± 10	87.6	84.8
CYP2C19 (70 μmol/L)^3^	9.11 ± 0.38	0.179 ± 0.008	50.9	17.5 ± 3.2	21.7 ± 0.8	0.59	3.9
CYP2E1 (87 μmol/L)^2^	228 ± 5	1.80 ± 0.02	126	52.1 ± 2.7	15.8 ± 0.3	4.3	7.0

Refer to the experimental section for probe substrates used for estimating the metabolic activity of particular CYP isoforms. The reaction velocity of 5-FU formation from *R*- and *S*-FT was determined using recombinant CYP isoforms as the enzyme source. Each value shown is the average of triplicate determinations (mean ± SD). RAF, CLint of CYPn in microsomes/CLint of cDNA-expressed CYPn; CYP contribution (%), RAF × (*V*_CYPn_)/(*V*_CYPn_) × 100 where *V* is the velocity in recombinant CYP-mediated conversion of FT to 5-FU. See also the Material and Methods for the detail.

1 Bourrié et al. (1996).

2 Bogarrds et al. (2000).

3 Coller et al. (1999).

## Discussion

It has been reported that two pathways exist in the bioactivation of FT, mainly in microsomes and partly in cytosol (El Sayed and Sadée 1982, 1983; Ikeda et al. 2000; Komatsu et al. 2000, 2001; Kajita et al. 2003). Therefore, we first investigated the contributions of CYP in microsomes and TPase in cytosol to the enantioselective conversion of FT to 5-FU using hepatic S9. The conversion of both FT enantiomers to 5-FU was observed in human hepatic S9, and *R*-FT was metabolized preferentially compared with *S*-FT. Furthermore, while 1-aminobenzotriazole, a nonspecific CYP inhibitor, exerted a strong inhibitory effect on the conversion of both FT enantiomers, TPI, a potent TPase inhibitor, had only a slight effect on the metabolism of *R*-FT. Inhibition of the metabolism of *S*-FT by TPI was not observed. These results suggest that CYP plays an important role in the enantioselective conversion of FT to 5-FU and the contribution of TPase is negligible in humans. This finding was supported by the kinetic data for the enantioselective metabolism of FT in human hepatic preparations. For example, hepatic cytosol showed the approximately 22-fold lower CLint value (0.29 μL min^−1^ mg^−1^) of *R*-FT than the total CLint value (6.4 μL min^−1^ mg^−1^) calculated as the sum of the CLint values in high- and low-affinity components for *R*-FT in microsomes.

Kinetic analysis for the enantioselective conversion of FT to 5-FU in human hepatic microsomes revealed that multiple CYP isoforms were involved in the metabolism of *R*-FT. The CLint value of the high-affinity component for *R*-FT (6.2 μL min^−1^ mg^−1^) was ∼27-fold higher than that of the low-affinity component (0.22 μL min^−1^ mg^−1^), which suggests that the high-affinity component is the main contributor (over 90%) to the conversion of *R*-FT to 5-FU when the concentration range of the substrate is well below the *K*_*m*_ value (0.12 mmol/L). On the other hand, the conversion of *S*-FT appears to occur in a monophasic manner with the *K*_*m*_ value (1.6 mmol/L). Although interindividual variation was observed in the enantioselective metabolism of FT by microsomes prepared from five human livers, respective microsomal preparations exhibited similar *K*_*m*_ values. This finding suggests that interindividual differences in the metabolic activity of human hepatic microsomes for FT are attributed to the variations in *V*_max_. Although the oral bioavailability of racemic FT after oral administration is approximately 100%, marked differences in the pharmacokinetic profiles of *R*- and *S*-FT are observed in cancer patients orally given FT (Damle et al. 2001). In the above study, the pharmacokinetic parameters of FT enantiomers were calculated from their plasma concentrations: that is, the area under the plasma concentration–time curve values of *R*- and *S*-FT were 10,800 and 49,300 ng h/mL, and the plasma elimination half-lives of *R*- and *S*-FT were 2.4 and 10.3 h, respectively, giving a higher oral clearance of *R*-FT compared with *S*-FT. In the present study, the CLint value of *R*-FT (6.4 μL min^−1^ mg^−1^) was 8.8-fold higher than that of *S*-FT (0.73 μL min^−1^ mg^−1^). Taken together, it would be highly likely that hepatic catalytic activity toward *R*- and *S*-FT is a major determinant of the plasma clearance in humans in vivo.

In this study, the following three approaches were used to reveal the relative contribution of CYP isoforms to the hepatic enantioselective metabolism of FT: (1) metabolism of probe substrates catalyzed by recombinant CYP isoforms as well as human hepatic microsomes; (2) inhibition studies with chemical inhibitors and inhibitory antibodies; and (3) estimation of the relative contribution of CYP isoforms to the metabolism based on RAF. These studies verified that CYP2A6 was a main contributor to bioactivation of both FT enantiomers. The enantioselectivity was clearly observed in the conversion of FT to 5-FU catalyzed by cDNA-expressed CYP isoforms. CYP2A6 is the most efficient catalyst for the metabolism of *R*-FT among CYP isoforms tested, and showed a *K*_*m*_ value of 0.17 mmol/L, which is close to the apparent *K*_*m*_ value of 0.12 mmol/L observed in the high-affinity component of human hepatic microsomes. On the other hand, the *K*_*m*_ values of CYP1A2 and 2E1 were determined to be 1.1 and 3.6 mmol/L, respectively. These values seem to match the apparent *K*_*m*_ value of 2.7 mmol/L in the low-affinity component of *R*-FT kinetics. These lines of evidence suggest that CYP2A6 plays an important role in the high-affinity clearance of *R*-FT in liver, and other CYP isoforms contribute to the low-affinity phase reactions. As compared with *R*-FT, *S*-FT metabolism showed a higher *K*_*m*_ value of 1.1 mmol/L, which results in a lower CLint value of 4.9 nL min^−1^ pmol^−1^ CYP in the metabolism mediated by CYP2A6. Also, CYP2C19 (a *K*_*m*_ value of 6.2 mmol/L) and CYP2E1 (a *K*_*m*_ value of 3.7 mmol/L) catalyzed the low-affinity reaction for *S*-FT. Based on these parameters, because of the involvement of several CYP isoforms with comparable *K*_*m*_ values, hepatic microsomes for the bioactivation of *S*-FT exhibited apparent monophasic kinetics. An inhibitory study using CYP-selective inhibitors and anti-CYP antibodies clarified the significant contribution of CYP2A6 to the enantioselective metabolism of FT. Tranylcypromine, a potent CYP2A6 inhibitor, inhibited 5-FU formation from both FT enantiomers by over 90%. Then, diethyldithiocarbamate, a CYP2E1 inhibitor, showed moderate inhibition, 27 and 34%, against 5-FU formation from *R*-FT and *S*-FT, respectively, and ticlopidine, a CYP2C19 inhibitor, slightly reduced the amount of 5-FU formed. Because disulfiram formed from diethyldithiocarbamate can efficiently inhibit CYP2A6 activity, on the basis of inhibition by diethyldithiocarbamate, the contribution of CYP2E1 may be overestimated (Ono et al. 1996). Similar trends to the data obtained using chemical inhibitors were observed in the reactions with anti-CYP antibodies. For example, polyclonal anti-CYP2A6 antibody strongly inhibited 5-FU formation from *R*- and *S*-FT catalyzed by human hepatic microsomes. However, in the case of other CYP isoforms, less than 10% inhibition against 5-FU formation from FT was observed even at the concentrations of antibodies up to 40 μL/mg microsomal protein. In accordance with the above estimation, RAF approach also suggested that CYP2A6 was the most important catalyst accounting for over 85% contribution to the enantioselective metabolism of FT. Conversely, the relative contribution of other CYP isoforms except for CYP2A6 was estimated to be less than 10%. 5-FU formation rates from *R*- and *S*-FT (30 μmol/L) in hepatic microsomes estimated using RAF values were 152 and 29 pmol min^−1^ mg^−1^, respectively, which seemed to be similar to the actual velocities (138 for *R*-FT and 21 pmol min^−1^ mg^−1^ for *S*-FT). Therefore, the percentage contribution of CYP isoforms estimated using RAF method is considered reasonable. The present study revealed that the conversion of both *R*- and *S*-FT to 5-FU was mainly catalyzed by the same CYP isoform, CYP2A6, in human liver at concentrations in the usual therapeutic range. The maximum concentrations (C_max_) of *R*- and *S*-FT in cancer patients after oral administration of UFT are 2957 (14.8 μmol/L) and 3788 ng/mL (18.9 μmol/L), respectively, which are much lower than their *K*_*m*_ values (Damle et al. 2001). Thereby, in humans in vivo, the metabolic interaction between FT enantiomers is thought not to occur when administered as the racemate.

Although there is a marked difference in CLint values between *R*- and *S*-FT, the role of each isomer as the precursor of 5-FU at the steady-state phase after the consecutive administration seems to be comparable, especially after coadministration with a DPD inhibitor such as CDHP. The concentration of 5-FU formed from *R*-FT rapidly increases in the plasma of cancer patients orally given racemic FT, and then reaches the C_max_. On the other hand, after repeated administration of racemic FT, *S*-FT accumulates in the plasma at the steady-state due to its lower metabolic clearance, which ensures that the plasma concentration of 5-FU remains at the sustained level. Thus, the prolonged plasma concentration of 5-FU plays an important role in the therapeutic efficacy after the coadministration of FT-based antitumor agents with a DPD inhibitor.

In conclusion, this study revealed that while 5-FU was generated from both *R*- and *S*-FT in human liver, their metabolic rates were significantly different. CYP2A6 was the main contributor to the bioactivation of both FT enantiomers to 5-FU, but metabolized *R*-FT more favorably than *S*-FT. Further, we observed the minor contribution of CYP1A2, CYP2C19, and CYP2E1 to the low-affinity phase metabolism of both isomers. These findings explain the higher plasma concentration of *S*-FT than that of *R*-FT in humans in vivo when racemate of FT is orally administered.

## Abbreviations

5-FU: 5-fluorouracil
CDHP: 5-chloro-24-dihydroxypyridine
CLint: intrinsic clearance
CYP: cytochrome P450
DPD: dihydropyrimidine dehydrogenase
ESI: electrospray ionization
FT: tegafur
IS: internal standard
LC/MS/MS: liquid chromatography-tandem mass spectrometry
MRM: multiple reaction monitoring
RAF: relative activity factor
S9: 9000*g* supernatant
TPase: thymidine phosphorylase
TPI: 5-chloro-6-(2-iminopyrrolidin-1-yl) methyl-24 (1*H*,3*H*)-pyrimidinedione hydrochloride
UFT: tegafur-uracil
β-NADP^+^: β-nicotinamide adenine dinucleotide phosphate
